# Arthroscopy Resources and Service Availability in Tanzania: A National Multidomain Assessment

**DOI:** 10.5334/aogh.5472

**Published:** 2026-11-13

**Authors:** Samih Issa, Kitugi Nungu, Joel Zacharia, Mohamed Muhamedhussein, Joseph Sabas, Francis Fabian, Adam Hussein, Allen Kisanga, Felix Mrita

**Affiliations:** 1 Department of Orthopaedics, Traumatology & Neurosurgery, Muhimbili University of Health and Allied Sciences, Dar es Salaam, Tanzania; 2 Department of Orthopaedics and Traumatology, Shifaa PanAfrican Hospital, Dar es Salaam, Tanzania; 3 Department of Orthopaedics and Traumatology, Muhimbili Orthopaedic Institute, Dar es Salaam, Tanzania

**Keywords:** arthroscopy, health services research, global surgery, health resources, low- and middle-income countries, Tanzania

## Abstract

*Background:* Arthroscopy requires specialised personnel, equipment, and reliable supplies. Resource deficiencies have been documented in sub-Saharan Africa, but national data from Tanzania are lacking.

*Objective:* To assess arthroscopy resources and service availability in Tanzanian hospitals providing specialist orthopaedic care and examine differences by hospital tier and ownership.

*Methods:* A national cross-sectional survey was conducted between January and February 2025 using orthopaedic surgeons as key informants to provide hospital-level data from their primary hospitals of practice. The hospital was the unit of analysis. Arthroscopy-related resources were assessed across five domains: personnel, infrastructure, procedures, equipment, and supplies. Overall resource availability was summarised using a study-specific Arthroscopy Resource Availability Score (ARAS) and compared across hospital tiers.

*Findings:* A total of 171 orthopaedic surgeons responded (response rate, 77.7%), providing data on 92 hospitals. Seven hospitals (7.6%) offered arthroscopy services, six of which were in Dar es Salaam. Arthroscopy surgeons were available in eight hospitals (8.7%). Although anaesthesia, rehabilitation, and supporting surgical infrastructure were widely available, 75.0% of hospitals lacked arthroscopy instruments. Arthroscopy-specific equipment, consumables, and implants were available in fewer than 15% of hospitals. Most facilities (83; 90.2%) had low resource availability (ARAS <50%). Median ARAS was 21.9% (IQR, 18.8–28.1), with higher scores in higher-tier than lower-tier hospitals (40.6% vs. 21.9%; *P* < 0.001). Hospitals offering arthroscopy were more likely to be higher-tier facilities (RR, 5.2; 95% CI, 2.6–10.4; *P* < 0.001).

*Conclusions:* Arthroscopy services were uncommon among the hospitals assessed and concentrated in higher-tier facilities, predominantly in Dar es Salaam. Planning service expansion should consider coordinated workforce development, equipment maintenance, reliable supplies, and institutional support. Phased development through selected referral centres warrants consideration, although its feasibility and effectiveness require further assessment.

## Introduction

Musculoskeletal disorders and injuries are major contributors to disability and create substantial demand for orthopaedic care [1, 2]. In low- and middle-income countries (LMICs), health systems often lack the surgical personnel, infrastructure, and equipment needed to meet this demand [3, 4]. Access to orthopaedic surgery remains uneven in sub-Saharan Africa, where resource shortages and limited referral capacity restrict treatment options [3–5]. In Tanzania, studies have documented a considerable burden of orthopaedic conditions and geographic barriers to surgical care [6, 7].

Arthroscopy allows the diagnosis and treatment of selected joint conditions through minimally invasive techniques. For appropriate indications, it may reduce soft tissue trauma, shorten hospital stays, and support earlier rehabilitation compared with open procedures [8, 9]. Establishing a service requires more than purchasing equipment: trained surgeons, appropriate instruments, functioning equipment, and reliable consumable supplies must be available together for procedures to be offered consistently [8–10].

Meeting these requirements remains difficult in many resource-constrained settings. Shortages and uneven distribution of surgical specialists compound limitations in arthroscopy training, equipment access, and operative exposure [11–14]. These constraints are interconnected: surgeons require regular practice to develop and maintain their skills, while hospitals need trained personnel to use equipment effectively. Experience from international partnerships also highlights the institutional support needed to sustain services beyond training visits or equipment donations [10]. Understanding existing resources and gaps is therefore necessary for planning service development.

Broader surgical assessments in LMICs have identified shortages across workforce, infrastructure, equipment, and supplies [15–19]. However, they do not establish whether hospitals possess the specific combination of resources required for arthroscopy. The Personnel, Infrastructure, Procedures, Equipment, and Supplies (PIPES) framework offers a structured approach to examining these components [20–23]. Adapting this approach to arthroscopy allows general surgical support to be assessed alongside specialised personnel, instruments, and consumables.

National data on arthroscopy resource and service availability in Tanzania are lacking. It remains unclear how widely services are offered, which resource gaps are most common, and how availability differs between hospital categories. This study therefore assessed arthroscopy-related resources and service availability across hospitals providing specialist orthopaedic care in Tanzania, using a PIPES-informed approach, and examined differences by hospital tier and ownership.

## Methods

### Study design and setting

This national cross-sectional survey was conducted in Tanzania between January and February 2025. It was a predefined arthroscopy-focused analysis of a broader national survey evaluating orthopaedic service delivery in Tanzania. Practising orthopaedic surgeons served as key informants and provided hospital-level data from their primary hospitals of practice.

The hospital was the unit of analysis. Data were collected using a structured, self-administered online questionnaire. All findings were based on surgeon-reported hospital data and were not independently verified through onsite facility assessment.

### Study population and sampling

Because arthroscopy is a specialised orthopaedic procedure, the sampling frame focused on hospitals providing specialist orthopaedic care. In the absence of a national registry identifying such hospitals, facilities were identified through practising orthopaedic surgeons registered with the Tanzania Orthopaedic Association (TOA), who reported on resources and services at their primary hospitals of practice.

Orthopaedic surgeons were selected as key informants because their specialist training and clinical practice provide familiarity with the personnel, infrastructure, equipment, instruments, and supplies required to support basic arthroscopy services. Hospitals across district, regional, zonal, and national referral levels were represented. All hospitals included in the final analysis employed at least one full-time orthopaedic surgeon.

### Data collection tools and measures

Data were collected using a structured questionnaire developed as part of a broader national assessment of orthopaedic service delivery in Tanzania. Arthroscopy-specific variables were organised into five domains informed by the PIPES framework. As no standardised instrument exists for assessing arthroscopy resources at the hospital level, tracer indicators were selected based on published literature, expert opinion, clinical relevance, and feasibility of surgeon-reported assessment. A total of 32 indicators were included across the 5 domains. Detailed descriptions of the questionnaire variables are provided in Supplementary File 1.

Resources and services were considered available only if reported to be consistently present, functional, and accessible for routine clinical use. Indicators were coded as binary variables, with available resources assigned a value of 1 and unavailable, non-functional, or inconsistently available resources assigned a value of 0.

The questionnaire underwent expert review by senior orthopaedic surgeons with experience in arthroscopy and surgical systems research to assess content validity. It was then pilot-tested among practising orthopaedic surgeons to evaluate clarity, relevance, and feasibility before electronic administration through Google Forms over six weeks.

### ARAS development and scoring

To facilitate comparison of arthroscopy resource availability across hospitals, the Arthroscopy Resource Availability Score (ARAS) was developed as a study-specific composite measure by summing available indicators and expressing the total as a percentage of the maximum achievable score. Each indicator was assigned equal weight because the assessment measured resource availability rather than quality, utilisation, or relative importance. ARAS was therefore interpreted as a summary measure of selected resource availability rather than a validated measure of hospital readiness, service capacity, or performance. Details of the ARAS domains, indicator selection, and scoring methodology are provided in Supplementary File 2.

### Variables of interest

The primary variables of interest were the availability of arthroscopy-related personnel, infrastructure, procedures, equipment, and supplies at the hospital level. Overall resource availability was summarised using ARAS. Additional variables included hospital tier, ownership category, geographic location, and arthroscopy procedural volume.

### Data management and statistical analysis

Survey data were exported to Microsoft Excel (Version 365) for anonymisation, cleaning, coding, and consolidation of duplicate hospital submissions. When multiple responses were received from the same hospital, submissions were reviewed for completeness and consistency, consolidated into a single hospital-level record, and discrepancies were clarified with respondents before finalising the dataset. Statistical analyses were performed using IBM SPSS Statistics (Version 26; IBM Corp., Armonk, NY, USA).

Categorical variables were summarised using frequencies and percentages, while ARAS was summarised using medians and interquartile ranges (IQRs). For descriptive purposes, hospitals were classified as having high (≥80%), moderate (≥50%–<80%), or low (<50%) resource availability based on ARAS. These study-defined thresholds were used for descriptive comparison and did not represent validated categories of arthroscopy resource availability.

For comparative analyses, hospitals were grouped as higher-tier (zonal and national) or lower-tier (district and regional) facilities and by ownership as public or non-public (private and faith-based/NGO). Comparisons were performed using Pearson chi-square test, Fisher exact test, Mann–Whitney U test, or Kruskal–Wallis test, as appropriate. Binary associations were reported as relative risks (RRs) with 95% confidence intervals (CIs). Statistical significance was defined as *P* < 0.05.

### Addressing potential sources of bias

All orthopaedic surgeons registered with the TOA were invited to participate. Repeated reminders and dissemination through multiple professional communication channels were used to encourage participation. To reduce reporting errors, the questionnaire underwent expert review and pilot testing. Duplicate hospital submissions were reviewed and consolidated, and discrepancies were clarified with respondents before analysis.

## Results

### Hospital characteristics and availability of arthroscopy services

A total of 171 orthopaedic surgeons participated in the survey, representing a response rate of 77.7% and providing data on 92 hospitals offering specialist orthopaedic care. Most hospitals were lower-tier facilities (72, 78.3%), and 44 (47.8%) were publicly owned. Arthroscopy services were available in only 7 (7.6%) of the assessed hospitals, 6 of which were located in Dar es Salaam.

### Arthroscopy-related personnel and infrastructure

Arthroscopy-competent surgeons were available in only 8 (8.7%) hospitals, and nurses trained in orthopaedic care in 45 (48.9%). In contrast, anaesthesia providers and rehabilitation personnel were available in 86 (93.5%) and 83 (90.2%) hospitals, respectively (Table 1).

**Table 1:** Surgeon-reported availability of arthroscopy-related personnel and supporting infrastructure in Tanzanian hospitals providing specialist orthopaedic care (*N* = 92).

VARIABLE	COUNT (*N*)	PERCENT (%)
**Personnel (Consistently Available)**		
Arthroscopy Surgeon	8	8.7
Anaesthesia Provider	86	93.5
Orthopaedic-Trained Nurse	45	48.9
Rehabilitation Provider	83	90.2
**Number of Arthroscopy Surgeons per Hospital**		
None	84	91.3
1	4	4.3
2–3	3	3.3
≥4	1	1.1
**Infrastructure (Consistently Available)**		
Exclusive Orthopaedic Theatre	61	66.3
Sterilisation Unit	78	84.8
Rehabilitation Unit	86	93.5
Plain Radiography	89	96.7
Magnetic Resonance Imaging	30	32.6

*Note:* Values are *n* (%), with percentages calculated using all 92 hospitals. Personnel and infrastructure were considered available when reported as consistently present and accessible for routine clinical use; infrastructure also had to be functional. Each hospital contributed one consolidated record.

Supporting infrastructure was more widely available than specialised arthroscopy personnel. Plain radiography, rehabilitation units, and sterilisation services were available in 89 (96.7%), 86 (93.5%), and 78 (84.8%) hospitals, respectively. Exclusive orthopaedic theatre space was available in 61 (66.3%) hospitals, whereas magnetic resonance imaging (MRI) was available in only 30 (32.6%) hospitals (Table 1).

### Arthroscopy procedures and procedural volume

Diagnostic arthroscopy, partial meniscectomy, debridement and microfracture, and anterior cruciate ligament reconstruction were available in all seven hospitals providing arthroscopy services. Meniscus repair, posterior cruciate ligament reconstruction, and shoulder arthroscopy procedures were less widely available (Table 2). Procedural volume was generally low; one hospital performed more than six arthroscopy procedures per week, five performed one to six procedures per week, and one performed one to three procedures per month.

**Table 2:** Surgeon-reported arthroscopy procedural volume and procedure availability in Tanzanian hospitals providing specialist orthopaedic care (*N* = 92).

VARIABLE	COUNT (*N*)	PERCENT (%)
**Arthroscopy Procedural Volume**		
None	85	92.4
1–3/month	1	1.1
1–3/week	4	4.3
4–6/week	1	1.1
>6/week	1	1.1
**Arthroscopy Procedures Available**		
Diagnostic Arthroscopy	7	7.6
Meniscus Repair	6	6.5
Partial Meniscectomy	7	7.6
Debridement and Microfracture	7	7.6
ACL Reconstruction	7	7.6
PCL Reconstruction	6	6.5
Rotator Cuff Repair	5	5.4
Subacromial Decompression	5	5.4

*Note:* Values are *n* (%), with percentages calculated using all 92 hospitals, including those not providing arthroscopy. Volume categories describe the reported frequency of arthroscopy procedures at each hospital. Procedure availability indicates procedures reported as consistently available for routine clinical care. Hospitals could offer more than one procedure. ACL, anterior cruciate ligament; PCL, posterior cruciate ligament.

### Arthroscopy equipment, instruments, and consumables

Arthroscopy-specific equipment was limited across hospitals, with arthroscopy towers, high-definition cameras, light sources, and shavers available in fewer than 15% of facilities. Irrigation pumps and radiofrequency ablation devices were even less frequently available. In contrast, supporting operative equipment, including electrical suction machines and intraoperative fluoroscopy, was more widely available (Table 3).

**Table 3:** Surgeon-reported adequacy of arthroscopy instruments and availability of equipment, consumables, and implants in Tanzanian hospitals providing specialist orthopaedic care (*N* = 92).

VARIABLE	COUNT (*N*)	PERCENT (%)
**Adequacy of Arthroscopy Instruments**		
Excellent	3	3.3
Good	8	8.7
Fair	6	6.5
Poor	6	6.5
Not Available	69	75.0
**Arthroscopy Equipment (Consistently Available)**		
Arthroscopy Tower	8	8.7
High-Definition Camera	8	8.7
Light Source	11	12.0
Shaver	9	9.8
Irrigation Pump	4	4.3
Radiofrequency Ablation	3	3.3
Electrical Suction Machine	84	91.3
Intraoperative Fluoroscopy	44	47.8
**Arthroscopy Consumables and Implants (Consistently Available)**		
Disposable Gowns and Drapes	13	14.1
Ethibond Sutures	12	13.0
Endobutton	7	7.6
Titanium Interference Screw	4	4.3
Fibertape	6	6.5
Bioabsorbable Screw	3	3.3
Meniscal Repair Suture	2	2.2

*Note:* Values are *n* (%), with percentages calculated using all 92 hospitals. Instrument adequacy categories reflect respondents’ ratings. Equipment, consumables, and implants were considered available when consistently present and accessible for routine clinical use; equipment also had to be functional. Resource items are reported separately, and percentages across items are not intended to sum to 100%.

Arthroscopy instruments were unavailable in 69 (75.0%) hospitals, and only 11 (12.0%) rated instrument adequacy as good or excellent. Arthroscopy-specific consumables and implants were similarly limited, with most items available in fewer than 10% of hospitals (Table 3).

### Arthroscopy resource availability score and comparative analysis

Overall, 83 (90.2%) hospitals were classified as having low resource availability (ARAS <50%). The median ARAS was 21.9% (IQR, 18.8–28.1). Higher-tier hospitals had significantly higher ARAS values than lower-tier hospitals (40.6% vs. 21.9%; *P* < 0.001).

Hospitals providing arthroscopy services were more likely to be higher-tier facilities (RR, 5.2; 95% CI, 2.6–10.4; *P* < 0.001). Six of the seven hospitals providing arthroscopy services were located in Dar es Salaam (RR, 2.7; 95% CI, 1.6–4.6; *P* = 0.006). No significant difference in ARAS was observed by hospital ownership (*P* = 0.259).

## Discussion

### Principal findings

Arthroscopy services were reported in 7 of the 92 hospitals assessed, 6 of which were in Dar es Salaam. Most hospitals had supporting services, such as anaesthesia, rehabilitation, sterilisation, and radiography, but few had arthroscopy surgeons or the specialised equipment and supplies needed to offer procedures routinely. Higher-tier hospitals had greater overall resource availability, while no significant difference in ARAS was observed by ownership. These findings suggest that, within the hospitals surveyed, the principal gaps concerned the specialised requirements of arthroscopy rather than the absence of basic surgical support.

Reports from other resource-constrained settings describe similar difficulties in establishing arthroscopy services, particularly in training personnel and maintaining access to equipment and consumables [8–12]. Broader surgical assessments in Rwanda, Bolivia, and Botswana also show why the presence of general infrastructure does not necessarily indicate that a hospital can provide a particular surgical service [14–16]. Our findings extend this evidence by identifying the resources available specifically for arthroscopy across Tanzanian hospitals providing specialist orthopaedic care.

### Workforce constraints and procedural activity

Arthroscopy surgeons were available in only eight hospitals. Procedural activity was also limited, with just one hospital reporting more than six arthroscopy procedures per week. Together, these findings raise questions about the opportunities available for surgeons to acquire and maintain arthroscopy skills. However, the survey cannot establish whether low activity resulted from workforce shortages, equipment limitations, referral patterns, treatment costs, or patient demand.

Babalola et al. [11] reported limited arthroscopy exposure and equipment access during orthopaedic training in a resource-constrained setting. Work on skills assessment likewise highlights the importance of evaluating practical skills alongside training exposure [12]. The wider shortage and uneven distribution of surgical specialists provide further context for these challenges [13]. For Tanzania, increasing the number of trained arthroscopy surgeons will therefore need to be considered alongside opportunities for supervised practice and the resources available at their hospitals.

### Equipment and consumable constraints

Most hospitals lacked arthroscopy instruments, and specialised equipment, implants, and consumables were uncommon. The wider availability of general operative equipment offers a potential foundation for service development, but does not establish that a hospital can sustain routine arthroscopy. A missing instrument or unavailable consumable may prevent a procedure even when other equipment is present.

Tibor and Hoenecke [8] emphasised the importance of maintenance and consumable replacement when introducing arthroscopy. Goldhaber et al. [9] and von Kaeppler et al. [10] also described the equipment, training, and institutional requirements of establishing services in resource-constrained settings. These reports support planning procurement around the continuing needs of a service. Although our findings identify shortages across several resource categories, they do not explain whether these arose from acquisition costs, maintenance difficulties, procurement arrangements, or other constraints.

### Geographic and tier-based disparities

Six of the seven hospitals offering arthroscopy were in Dar es Salaam, and higher-tier hospitals had higher resource scores than lower-tier facilities. This distribution is consistent with the concentration of specialised surgical resources in referral centres described in other resource-constrained settings [5, 15–19]. The pattern may reflect the location of trained personnel, investment, and referral services, although these explanations were not directly examined.

Concentrating scarce expertise and equipment in selected hospitals may help maintain operative activity and support training. However, this arrangement may also create additional travel and referral requirements for patients living elsewhere. Because the study did not measure travel time, affordability, unmet need, or referral completion, the implications for patient access cannot be quantified from these data.

### Multidomain resource availability

The median ARAS was 21.9%, and 90.2% of hospitals were classified as having low resource availability. These results indicate that most hospitals reported only a small proportion of the selected indicators. Assessing several domains together provided information that would have been missed by considering infrastructure alone, an issue also relevant to broader surgical resource assessments [15, 18].

ARAS nevertheless requires cautious interpretation. It is a study-specific score with equally weighted indicators and has not been externally validated. Hospitals with similar scores may have different combinations of resources, and the absence of an essential item may matter more for service delivery than the total score suggests. ARAS should therefore be interpreted alongside the individual resource findings, rather than used as a measure of hospital readiness, quality, or performance.

### Implications for service development

The findings can help identify hospitals where specific investments might support arthroscopy provision. One option is phased development through selected referral centres that already have supporting surgical infrastructure. Such an approach could bring training, equipment, and supply arrangements together, but its feasibility would depend on local needs, referral pathways, and the ability to sustain procedural activity. This study did not compare alternative models of service expansion or assess their costs.

Workforce initiatives could incorporate supervised operative experience, mentorship, simulation, and institutional partnerships [10–12]. Equipment investment would similarly require arrangements for maintenance, instrument replacement, and continuing consumable supplies [8–10]. Organisational research in LMICs also suggests that leadership, teamwork, and institutional learning deserve attention when developing surgical services, although these factors were not assessed here [24].

These considerations relate to the workforce, infrastructure, equipment, and referral priorities discussed in Tanzania’s National Surgical, Obstetric, and Anaesthesia Plan [25]. The present findings provide baseline information for planning, but decisions about expansion should also consider population need, affordability, and competing demands on surgical resources.

### Future research

Onsite assessments are needed to verify reported availability and examine whether resources remain functional and accessible over time. Research on patient need, treatment costs, and referral pathways would help establish where additional services are most needed. Longitudinal studies could then assess changes following service-development initiatives. Further work should also evaluate the validity, reliability, and interpretation of ARAS before its wider application.

### Strengths and limitations

To our knowledge, this is the first national assessment of arthroscopy resource and service availability in Tanzania. The survey achieved a 77.7% surgeon response rate and provided information on 92 hospitals. Consolidating duplicate submissions allowed analysis at hospital level, while the PIPES-informed approach covered several components required for arthroscopy provision.

The study has several limitations. Resources were reported by surgeons and were not independently verified through onsite assessment, allowing the possibility of reporting inaccuracies. Hospitals were identified through orthopaedic surgeons registered with the TOA; facilities without an orthopaedic surgeon and those not represented by respondents were therefore outside the assessment. The findings should consequently be interpreted within the surveyed specialist orthopaedic hospital network rather than as a complete inventory of Tanzanian hospitals. ARAS and its classification thresholds are study-specific and have not been externally validated. Finally, the cross-sectional design could not establish causes of resource shortages or changes over time, and the survey did not assess patient access, affordability, service quality, or clinical outcomes.

## Conclusion

Arthroscopy services were available in a few of the Tanzanian hospitals assessed and were concentrated in higher-tier facilities, predominantly in Dar es Salaam. Although supporting surgical infrastructure was widely available, specialised personnel, equipment, instruments, and consumables were uncommon. Service planning should consider coordinated workforce development, equipment maintenance, and reliable supply systems. Sustained institutional support and phased development through selected referral centres may help expand provision, although the feasibility and effectiveness of this approach require further assessment.

## Data Availability

The datasets generated and analysed during the current study are available from the corresponding author upon reasonable request.

## Author Data Access and Manuscript Responsibility

All authors had access to the study data and participated in the preparation, review, or revision of the manuscript. All authors approved the final version of the manuscript.

## Additional Files

The additional files for this article can be found as follows:

Supplementary File S1. Arthroscopy Questionnaire Variables and Operational Definitions.

Supplementary File S2. Arthroscopy Resource Availability Score (ARAS): Domain Structure, Indicator Selection, and Scoring Methodology.
