# Supplementary material for: Arthroscopy Resources and Service Availability in Tanzania: A National Multidomain Assessment
Source: Ann Glob Health. 2026 Nov 13;92(1):115. doi: 10.5334/aogh.5472 (PMC13761850; doi:10.5334/aogh.5472)
Supplement: Supplementary file 1 [file agh-92-1-5472-s1.pdf]

Supplementary File 1. Arthroscopy Questionnaire Variables and Operational Definitions

A. *Hospital Characteristics*

1. Hospital name

2. Regional location

3. Facility tier
   - District hospital
   - Regional hospital
   - Zonal hospital
   - National hospital

4. Ownership category
   - Public
   - Private
   - Faith-based/NGO

5. Availability of arthroscopy services?
   - Yes
   - No

B. *Personnel Available*

1. Arthroscopy-competent surgeon
   - Yes
   - No
2. Anaesthesia provider
   - Yes
   - No
3. Orthopaedic-trained nurse
   - Yes
   - No
4. Physiotherapist/rehabilitation provider
   - Yes
   - No

Number of arthroscopy-competent surgeons available:
   - None
   - 1
   - 2–3
   - ≥4

C. *Infrastructure Available*

1. Exclusive functional orthopaedic theatre
   - Yes
   - No

2. Sterilisation unit
   - Yes
   - No

3. Physiotherapy/Rehabilitation unit (Physiotherapy)
   - Yes
   - No

4. Plain radiography unit
   - Yes
   - No

5. Magnetic resonance imaging (MRI) unit
   - Yes
   - No

D. *Procedures Available*

1. Diagnostic arthroscopy
   - Yes
   - No
2. Meniscus repair
   - Yes
   - No
3. Partial meniscectomy
   - Yes
   - No
4. Debridement and microfracture
   - Yes
   - No
5. ACL reconstruction
   - Yes
   - No
6. PCL reconstruction
   - Yes
   - No
7. Rotator cuff repair
   - Yes
   - No
8. Subacromial decompression
   - Yes
   - No

Arthroscopy procedural volume
- None
- 1–3/month
- 1–3/week
- 4–6/week
- >6/week

E. Equipment Available

1. Arthroscopy tower
   - Yes
   - No
2. High-definition camera
   - Yes
   - No
3. Light source
   - Yes
   - No
4. Shaver system
   - Yes
   - No
5. Irrigation pump
   - Yes
   - No
6. Radiofrequency ablation device
   - Yes
   - No
7. Electrical suction machine
   - Yes
   - No
8. Intra-operative fluoroscopy
   - Yes
   - No

Adequacy of arthroscopy instruments
- Excellent
- Good
- Fair
- Poor
- Not available

F. ***Supplies Available (Consumables)***

1. Disposable gowns and drapes
   - Yes
   - No
2. Ethibond sutures
   - Yes
   - No
3. Endobutton
   - Yes
   - No
4. Titanium interference screw
   - Yes
   - No
5. Fibertape
   - Yes
   - No
6. Bioabsorbable interference screw
   - Yes
   - No
7. Meniscal repair suture
   - Yes
   - No
   -

Supplementary Notes

The variables presented in this supplementary file were extracted from a broader national survey of orthopaedic service delivery in Tanzania and represent those used in the present arthroscopy-focused analysis. Variables assessed personnel, infrastructure, procedures, equipment, supplies, and service characteristics relevant to arthroscopy care at the hospital level.

All variables were based on surgeon-reported hospital data. Resources and services were considered available if reported as consistently present, functional, and accessible for routine clinical use at the time of data collection.
