# Supplementary material for: Arthroscopy Resources and Service Availability in Tanzania: A National Multidomain Assessment
Source: Ann Glob Health. 2026 Nov 13;92(1):115. doi: 10.5334/aogh.5472 (PMC13761850; doi:10.5334/aogh.5472)
Supplement: Supplementary file 2 [file agh-92-1-5472-s2.pdf]

Supplementary File 2. Arthroscopy Resource Availability Score (ARAS): Domain Structure, Indicator Selection, and Scoring Methodology

Supplementary Table 1*. *ARAS Domain Structure and Indicators

Domain	List of Indicators	Number of Indicators
Personnel	Arthroscopy-competent surgeon<br>Anaesthesia provider<br>Orthopaedic-trained nurse<br>Physiotherapy/rehabilitation provider	4
Infrastructure	Exclusive orthopaedic theatre<br>Sterilisation unit<br>Physiotherapy/rehabilitation unit<br>Plain radiography unit<br>Magnetic Resonance Imaging unit	5
Procedures	Diagnostic arthroscopy<br>Meniscus repair<br>Partial meniscectomy<br>Debridement and microfracture<br>ACL reconstruction<br>PCL reconstruction<br>Rotator cuff repair<br>Subacromial decompression	8
Equipment	Arthroscopy tower<br>High-definition camera<br>Light source<br>Shaver system<br>Irrigation pump<br>Radiofrequency ablation device<br>Electrical suction machine<br>Intra-operative fluoroscopy	8
Supplies	Disposable gowns and drapes<br>Ethibond sutures<br>Endo-button<br>Titanium interference screw<br>Bio-absorbable interference screw<br>Fiber-tape<br>Meniscal repair suture	7

Footnote: The maximum achievable ARAS was 32, derived from 32 equally weighted indicators across the five domains.

Supplementary Notes

Indicator Selection

The Arthroscopy Resource Availability Score (ARAS) was developed as a study-specific composite measure of arthroscopy resource availability at the hospital level. Indicators were selected based on expert opinion, clinical relevance, published literature, and feasibility of surgeon-reported assessment. Only indicators that could be objectively classified as available and functional or unavailable were included.

ARAS Calculation and Weighting

All indicators were coded as binary variables (available and functional = 1; unavailable, non-functional, or inconsistently available = 0). ARAS was calculated by summing available indicators across the five domains and expressing the total as a percentage of the maximum achievable score of 32. Each indicator was assigned equal weight because the assessment measured resource presence rather than quality, utilisation, or relative importance.

Variables Excluded from ARAS

Several collected variables were excluded from ARAS because they reflected resource quantity, service utilisation, subjective assessment, or service outputs rather than resource availability. These included the number of arthroscopy-competent surgeons, arthroscopy procedural volume, and adequacy of arthroscopy instruments.

Interpretation of ARAS

ARAS has not undergone formal external validation and should be interpreted as a study-specific composite measure of resource availability rather than a validated measure of hospital readiness, service capacity, performance, or quality.
